# Fault-Tolerant Control Based on Current Space Vectors against Total Sensor Failures

**DOI:** 10.3390/s24113558

**Published:** 2024-05-31

**Authors:** Cuong Dinh Tran, Martin Kuchar, Vojtech Sotola, Phuong Duy Nguyen

**Affiliations:** 1 Power System Optimization Research Group, Faculty of Electrical and Electronics Engineering, Ton Duc Thang University, Ho Chi Minh City 700000, Vietnam; 2 Department of Applied Electronics, Faculty of Electrical Engineering and Computer Science, VSB-Technical University of Ostrava, 708 00 Ostrava, Czech Republic; martin.kuchar@vsb.cz (M.K.); vojtech.sotola@vsb.cz (V.S.); phuong.nguyen.duy.st@vsb.cz (P.D.N.); 3 Faculty of Electronics and Telecommunication, Saigon University, Ho Chi Minh City 700000, Vietnam

**Keywords:** current space vector, estimated signal, fault-tolerant control, induction motor, sensor failure diagnosis

## Abstract

This paper proposes a fault-tolerant control (FTC) strategy using the current space vectors to diagnose sensor failures and enhance the sustained operation of a field-oriented (FO) controlled induction motor drive (IMD). Three space vectors are established for the sensor fault diagnosis technique, including one converted from the measured currents and the other two calculated from the current estimation technique, respectively, measured and with reference speeds. A mixed mathematical model using three space vectors and their components is proposed to accurately determine the fault condition of each sensor in the motor drive. After determining the operating status of each sensor, if the sensor signal is in good condition, the feedback signal to the controller will be the measured signal; otherwise, the estimated signal will be used instead of the failed signal. Failure states of the various sensors were simulated to check the effectiveness of the proposed technique in the Matlab/Simulink environment. The simulation results are positive: the IMD system applying the proposed FTC technique accurately detected the failed sensor and maintained stability during the operation.

## 1. Introduction

The three-phase induction motor (IM) has outstanding advantages of size, durability, stable operation in harsh environments, low cost, and a large production scale. Thus, this motor type is widely used in various industrial applications. In the last century, induction motors (IMs) were generally used for fixed-speed applications. Recently, in parallel with the development of power electronic technologies, the IMD system based on modern control algorithms such as Field-Oriented Control (FOC), Direct Torque Control (DTC), etc., has been extensively involved in precise speed control applications [1,2]. During operation, the IMD system requires feedback signals from current and speed sensors to perform control effectively and accurately. These sensors provide essential information about the position and operating condition of the motor, so if these sensors are damaged, the incorrect feedback provided will result in a reduced performance of the IMD system [3].

In industrial processes, many faults occur during operation that degrade the entire system’s performance. Modern methods rely on collecting abnormal data and feature-training processes such as multivariate statistical methods, machine learning methods, wide convolutional neural networks, etc., which are commonly used to diagnose various types of faults in industrial processes [4]. However, in the practical operation of IMD systems, the most severe fault is total failure, which occurs with the sensors integrated into the control system; thus, many studies have been conducted on sensor fault-tolerant control corresponding to total failure in recent times. In [5], a decision unit using the Extended Kalman filter is proposed to implement the FTC function for IM drives. Adaptive observers detect fault conditions and implement solutions to ensure that the system works even when sensor failures occur. The authors in [6] propose a sensor fault control strategy based on a Luenberger Observer (LO) combining axes transformation methods to detect the current sensor faults. When sensor failure is detected, the missing current information is replaced by the estimated current. However, it should be noted that this method does not consider the influence of the speed sensor on the estimated current in the current sensor fault diagnosis algorithm. A diagnosis algorithm based on a slip-independent estimated current combining a third difference operator to detect current faults is proposed in [7]. The advantage of this diagnosis method is the independence of the machine model, and it can also be applied to partial current sensor faults. In Ref. [8], a structural analysis method is applied to diagnose the sensor faults; the Dulmage Mendelsohn decomposition technique is used in the diagnosis model, according to the dynamic model in the matrix form. The authors in [9] present an FTC technique based on electrical torque to reduce the IMD system’s current sensor noise. This method enhances the reliability and performance of the drive in terms of the speed response, noise, and harmonics of the stator current. In [10], a current sensor FTC that does not use a virtual speed signal is proposed to detect the faulty sensor. The enhanced locked loop is applied to reconstruct the incorrectly measured current in each phase. In [11], a current sensor FTC based on the vector control technique and LO is proposed to detect the faults and then a logic circuit is used to switch to a proper current signal. Other FTC methods, as discussed in [12], involve control tuning based on the specific faulty sensor. Under normal operating conditions, direct torque control is used, while indirect field directional control is used when the DC-link voltage sensor malfunctions; if the current or speed sensor is broken, the scalar strategy is used to implement the speed control. Reference [13] presents an FTC scheme using the sliding mode observer with combined single phase enhanced phase-locked loop (SEPLL) against current sensor failures. After the incorrectly measured signals are determined, SEPLL signals reconfigure the false signals. In articles [14,15], current sensor diagnosis methods based on an integration algorithm [14] and based on the rotor slip [15] are developed to detect current sensor faults in the IMD during the operation.

The loss of a feedback signal due to the complete failure of the sensors or abnormal disconnection is a severe problem; it can cause the total collapse of the drive system. There are many groups of FTC methods used to diagnose sensor failures. However, for total failures, the most typical FTC technique is the method that uses virtual signals of estimators and observers for the comparison algorithm between virtual signals and measured signals to detect sensor faults. The disadvantage of this diagnosis technique is that the estimated current is generated from the measured rotor speed, and the estimated speed is also calculated from the measured current. Therefore, methods for diagnosing sensor faults based only on the comparison algorithm between the measured and estimated signals only apply to one current or speed sensor fault type.

This paper focuses on the diagnosis strategy of FTC using the current space vectors’ comparison algorithms to determine the signal’s operation states from sensors. The proposed method can diagnose total sensor faults for both current and speed sensors. When the measured signals are correct, they will be transferred to the FOC loop for speed control. If the sensor fails, faulty measured signals are rejected and replaced with estimated speed [16,17,18,19,20] and virtual currents [21,22,23,24,25]. The total failure states of the sensors are simulated to evaluate the effectiveness of the proposed technique.

## 2. Fault-Tolerant Control Strategy against the Sensor Faults

### 2.1. Mathematical Model of a Three-Phase Induction Motor

The relationships between the current, voltage, and flux quantities in an IM are based on a system of first-order differential equations. The relationship between the stator current components and the rotor flux components corresponding to the input voltage and machine parameters is shown by the following differential equation in the [*α*, *β*] stationary coordinate (mathematical symbols are explained in Table A1 in Appendix A):

(1) $$\frac{di_{S\alpha }}{dt}=-K_{1}i_{S\alpha }+K_{2}\Psi_{R\alpha }+K_{3}\omega_{r}\Psi_{R\beta }+K_{4}u_{S\alpha },$$

(2) $$\frac{di_{S\beta }}{dt}=-K_{1}i_{S\beta }+K_{2}\psi_{R\beta }-K_{3}\omega_{r}\psi_{R\alpha }+K_{4}u_{S\beta },$$

(3) $$\frac{d\psi_{R\alpha }}{dt}=K_{5}i_{S\alpha }-K_{6}\psi_{R\alpha }-\omega_{r}\psi_{R\beta },$$

(4) $$\frac{d\psi_{R\beta }}{dt}=K_{5}i_{S\beta }-K_{6}\psi_{R\beta }-\omega_{r}\psi_{R\alpha },$$

where

$$\begin{array}{l} K_{1}=\frac{R_{S}L_{R}^{2}+R_{R}L_{m}^{2}}{L_{S}L_{R}^{2}\sigma };\;K_{2}=\frac{R_{R}L_{m}}{L_{S}L_{R}^{2}\sigma };\;K_{3}=\frac{L_{m}}{L_{S}L_{R}\sigma };\\ K_{4}=\frac{1}{L_{S}\sigma };\;K_{5}=\frac{R_{R}L_{m}}{L_{R}};\;K_{6}=\frac{R_{R}}{L_{R}};\;\sigma =\frac{L_{S}L_{R}-L_{m}^{2}}{L_{S}L_{R}} \end{array}$$

**Figure 1:**
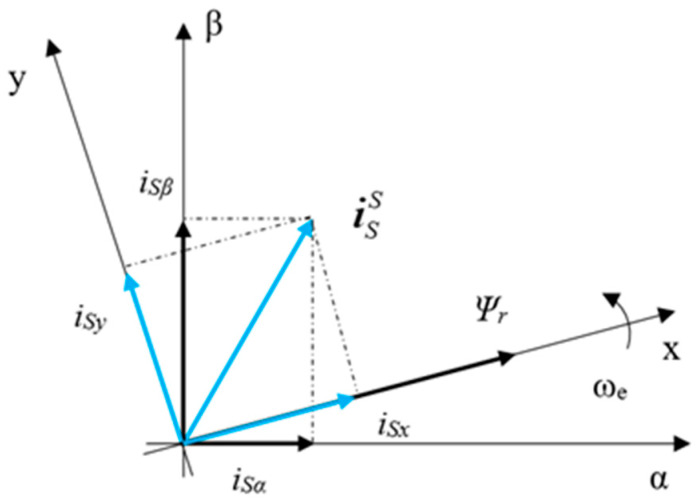
The current space vector corresponds to the FOC strategy.

In the past, the operating speed of the IM was determined as a value of (less than) a rated speed corresponding to a rotor slip. Overcoming the limitations of classical control methods, modern control methods such as DTC and FOC are often used to meet the increasing demand for precise speed control in IM applications. The research model of IMD in this paper applies the FOC strategy for motor speed control. In the FOC method, a rotation coordinate [*x*, *y*] with the *x*-axis equivalent to the rotor flux is used to separate the stator current into two perpendicular components, *i_Sx_* and *i_Sy_*, as shown in Figure 1 [15]. The current component *i_Sx_* will be controlled to maintain the rotor flux constant with the motor’s rated flux. On the other hand, the *i_Sy_* current component is used to control the motor speed relative to the setting value.

### 2.2. Fault-Tolerant Control

**Figure 2:**
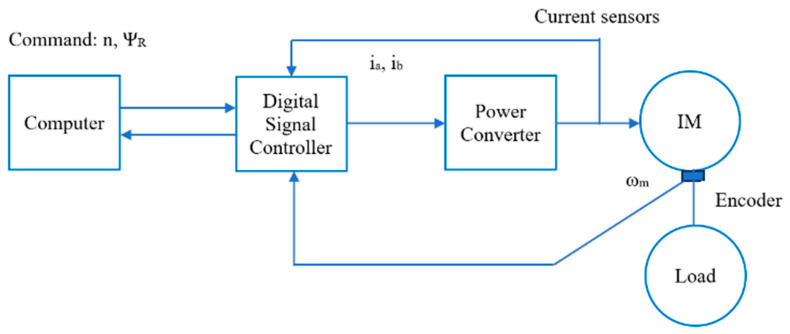
Control structure of the IMD.

**Figure 3:**
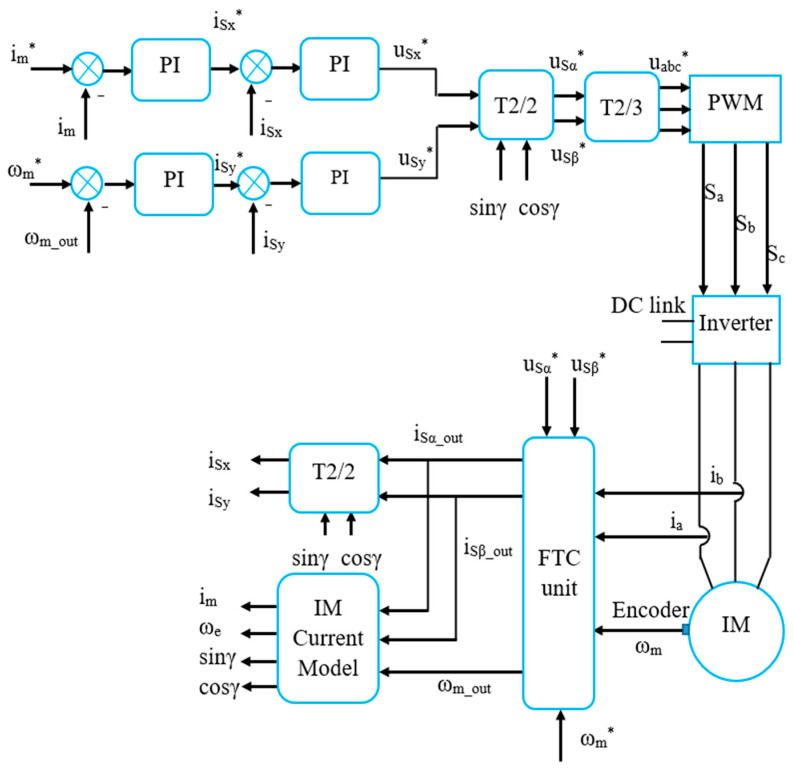
Detailed model of the IMD applying the FOC technique integrated with the FTC function.

The general structure of the IMD includes the following main parts: a motor connected to the load, a power converter, a Digital Signal Controller (DSC), a computer, and the sensors corresponding to Figure 2. A detailed model of the IMD systems applying the FOC method integrating FTC functions against sensor faults is illustrated in Figure 3.

**Figure 4:**
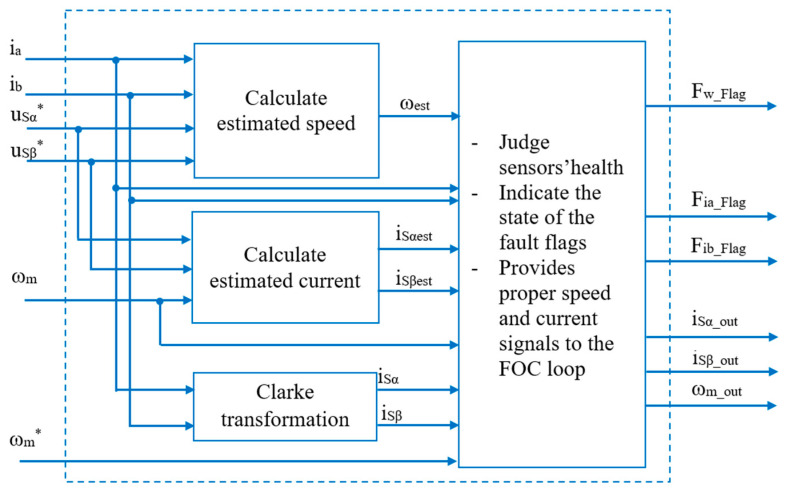
FTC unit.

The FTC will receive feedback signals from the sensor, such as current and speed, and will then conduct quality checks of these signals. If the signal matches, the sensor is healthy; the FTC will provide a measured signal to the FOC loop. Otherwise, the FTC will give a fault warning and provide the proper estimated signal to the speed controller. Figure 4 shows the overall block diagram of the FTC unit, including two signal estimation blocks and the feedback signal state diagnosis block.

The stator currents in the real-time domain will be transformed into space vectors in [*α*, *β*] stationary coordinates by Clarke transform. Current and voltage signals using estimation algorithms such as SMO, RFMRAS, CBMRAS, etc., refs. [16,17,18,19,20] will be applied to create an estimated speed to diagnose sensor conditions and replace the measuring speed if a speed sensor fault occurs. The voltage and measured speed signals are used to estimate the virtual current through proper estimation methods. In various estimation algorithms, the Luenbeger observer (LO) [25] is an appropriate method less affected by machine parameters; therefore, it is applied in this paper.

The sensor fault diagnosis algorithm is based on the measured and estimated current components in the [*α*, *β*] coordinate and the motor speeds, including measured, estimated, and reference speeds, to give status indications of the sensor signal while providing the proper current and speed for the FOC loop. Three current space vectors are created from the input signals. The first current vector based on the measured current signal is converted by Clarke transformation, and the magnitude of the vector is formed by the square root of the squares of the components (5):

(5) $$\left\{\begin{array}{l} \left[\begin{array}{l} i_{S\alpha }\\ i_{S\beta } \end{array}\right]=\left[\begin{array}{l} \;\;1\;\;\;\;\;\;\;0\\ \frac{1}{\sqrt{3}}\;\;\;\;\frac{2}{\sqrt{3}} \end{array}\right]\left[\begin{array}{l} i_{a}\\ i_{b} \end{array}\right]\\ I_{spm}=\sqrt{i_{S\alpha }{}^{2}+i_{S\beta }{}^{2}} \end{array}\right.,$$

The LO [25], according to Equations (6)–(9), is used to calculate the components of the current space vector in the [*α*, *β*] coordinate. The current space vector is determined according to the estimated values if the feedback speed is applied in the LO. Otherwise, if the reference speed is used in LO, the current space vector corresponds to the reference values:

(6) $$\frac{di_{S\alpha }}{dt}=-\frac{\left(L_{R}^{2}R_{S}+L_{m}^{2}R_{R}\right)}{L_{S}L_{R}^{2}\sigma }i_{S\alpha }+\frac{L_{m}R_{R}}{L_{S}L_{R}^{2}\sigma }\psi_{R\alpha }+\frac{L_{m}p\omega_{m}}{L_{S}L_{R}\sigma }\psi_{R\beta }+\frac{u_{S\alpha }^{*}}{L_{S}\sigma }-L_{1}i_{S\alpha }+L_{2}i_{S\beta },$$

(7) $$\frac{di_{S\beta }}{dt}=-\frac{\left(L_{R}^{2}R_{S}+L_{m}^{2}R_{R}\right)}{L_{S}L_{R}^{2}\sigma }i_{S\beta }-\frac{L_{m}p\omega_{m}}{L_{S}L_{R}\sigma }\psi_{R\alpha }+\frac{L_{m}R_{R}}{L_{S}L_{R}^{2}\sigma }\psi_{R\beta }+\frac{u_{S\beta }^{*}}{L_{S}\sigma }-L_{1}i_{S\beta }-L_{2}i_{S\alpha },$$

(8) $$\frac{d\psi_{R\alpha }}{dt}=\frac{L_{m}R_{R}}{L_{R}}i_{S\alpha }-\frac{R_{R}}{L_{R}}\psi_{R\alpha }-p\omega_{m}\psi_{R\beta }-L_{3}i_{S\alpha }+L_{4}i_{S\beta },$$

(9) $$\frac{d\psi_{R\beta }}{dt}=\frac{L_{m}R_{R}}{L_{R}}i_{S\beta }+p\omega_{m}\psi_{R\alpha }-\frac{R_{R}}{L_{R}}\psi_{R\beta }+L_{3}i_{S\beta }-L_{4}i_{S\alpha },$$

where

$$\begin{array}{l} L_{1}=(k-1)(\frac{1}{\sigma T_{S}}+\frac{1}{\sigma T_{R}});\;L_{2}=-(k-1)p\omega_{m};\\ L_{3}=(k^{2}-1)\left[(\frac{1}{\sigma T_{S}}+\frac{1}{\sigma T_{R}})\frac{\sigma L_{S}L_{m}}{L_{R}}-\frac{L_{m}}{T_{R}}\right]+\frac{\sigma L_{S}L_{m}}{L_{R}}(\frac{1}{\sigma T_{S}}+\frac{1}{\sigma T_{R}})(k-1);\\ L_{4}=-(k-1)\frac{\sigma L_{S}L_{m}}{L_{R}}p\omega_{m};T_{S}=\frac{L_{S}}{R_{S}};T_{R}=\frac{L_{R}}{R_{R}};\;\\ k>1\;(\mathrm{proportionality}\;\mathrm{factor},\;\mathrm{slightly}\;\mathrm{larger}\;\mathrm{than}\;“1”); \end{array}$$

Based on LO equations, the amplitude of the second current vector based on the virtual current signal and the amplitude of the third current vector based on the reference speed are calculated by (10) and (11):

(10) $$I_{spe}=\sqrt{i_{S\alpha est}{}^{2}+i_{S\beta est}{}^{2}},$$

(11) $$I_{spref}=\sqrt{i_{S\alpha ref}{}^{2}+i_{S\beta ref}{}^{2}},$$

To clarify, *I_spm_* depends only on the measured signal from the two current sensors, *I_spe_* depends on the feedback signal of the speed sensor, and *I_spref_* depends on the reference speed.

First, *I_spm_* and *I_spe_* are used to determine whether a sensor fault has occurred, as in Formula (12):

(12) $$\left\{\begin{array}{l} {\mathrm{Index}}_{F}=|I_{spm}-I_{spe}|\\ {\mathrm{If}\;(\mathrm{Index}}_{F}>Th_{F})\{F_{F\_Flag}=1;\} \end{array}\right.,$$

Then, the three modules of the three current vectors are compared in pairs to determine the sensor fault types:

(13) $$\left\{\begin{array}{l} {\mathrm{Index}}_{i}=|I_{spref}-I_{spm}|\\ \mathrm{If}\;(F_{F\_Flag}==1)\\ \left\{{\mathrm{If}\;(\mathrm{Index}}_{i}>Th_{i})\{F_{i\_Flag}=1;\}\;else\;\{F_{w\_Flag}=1;\}\right\} \end{array}\right.,$$

**Figure 5:**
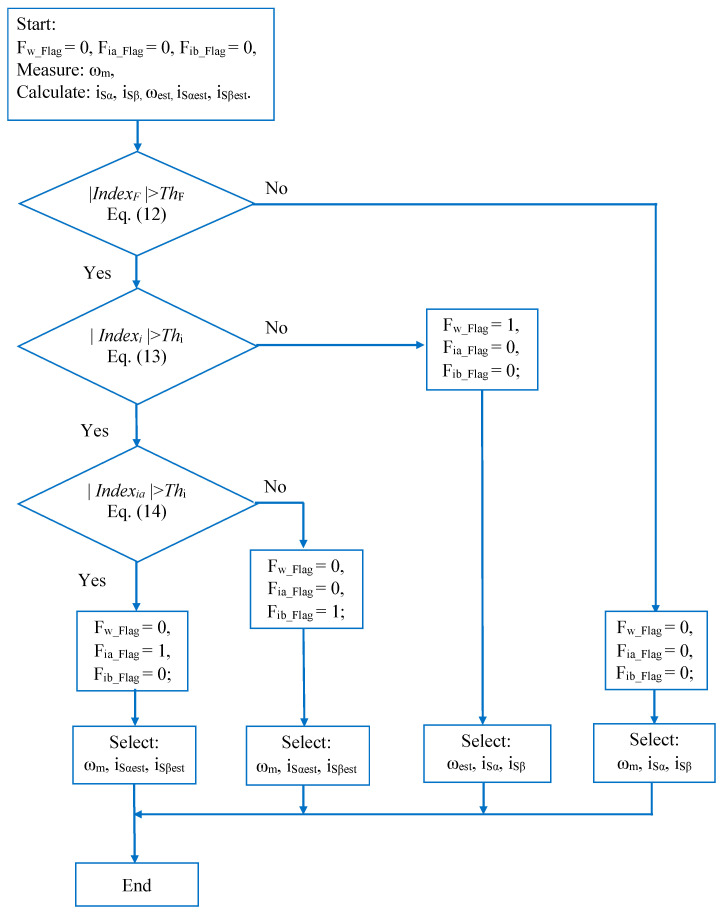
Flowchart of the proposed sensor fault diagnosis method.

In regular operation, the fault flag will be low (zero value); when a sensor fault occurs, the fault flag will rise to high (value one). We can determine the type of sensor failure based on formulas (13). If a current fault occurs, the precise phase of the current sensor that is faulty must be defined. Because the *I_Sα_* component in the current space vector corresponds to *i_a_* in the coordinate system [*a*, *b*, *c*], a comparison of the two components *I_Sα_* of the current space vector *I_spm_* and *I_spref_* is used to determine the faulty current phase, as in (14):

(14) $$\left\{\begin{array}{l} {\mathrm{Index}}_{ia}=|I_{S\alpha }-I_{S\alpha ref}|\\ {\mathrm{If}\;(F}_{i\_Flag}==1)\\ \left\{{\mathrm{If}\;(\mathrm{Index}}_{ia}>Th_{i})\{F_{ia\_Flag}=1;\}\;else\;\{F_{ib\_Flag}=1;\}\right\} \end{array}\right.,$$

where *Th_F_* and *Th_i_* are all the maximum deviations of the current space vectors under normal operation conditions; these thresholds correspond to 10% of the rated current value, which is reasonable (refer to research papers and performed simulations). The sequence of the sensor fault diagnosis method is presented in the flowchart in Figure 5.

As a result, it is possible to accurately diagnose each sensor’s health status and then decide on the corresponding operating mode. If the sensor status is good, the IMD system will operate in sensor mode; otherwise, when a sensor fault occurs, IMD will operate in sensorless mode. The control law, fault flag status, and corresponding output signal are shown in Table 1.

## 3. Simulation Results

**Figure 6:**
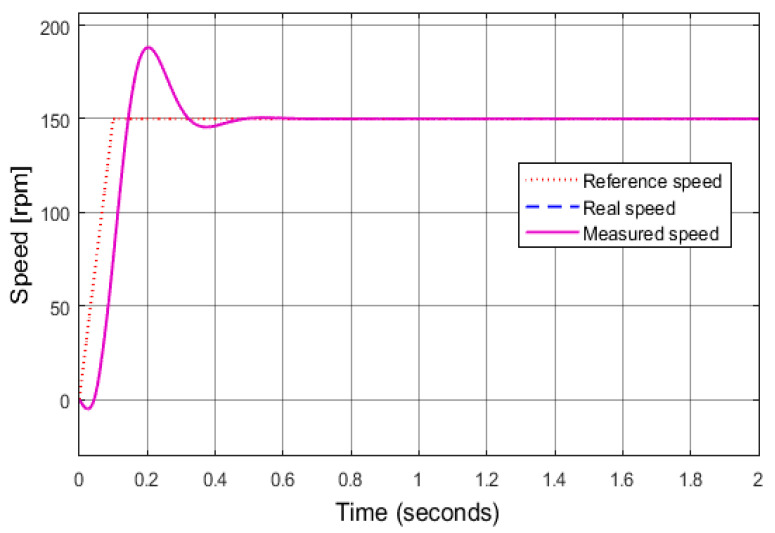
Reference, real, and measured speed in the IMD.

The proposed diagnostic technique based on current space vectors against the sensor failures is simulated in a Matlab/Simulink environment. The total failures according to three sensors are implemented to demonstrate the efficiency of the diagnostic methods. All motor parameters set for the simulation are listed as follows: rated power = 2.2 kW, rated speed = 1420 rpm, number of pole pairs *p* = 2, stator/rotor resistance = 3.179/2.118 Ω, and stator/rotor/mutual inductance = 0.192 h. Four operating modes of the drive are simulated, corresponding to a reference speed of 150 rpm, as shown in Figure 6. Where motor operation corresponds to the healthy mode of all sensors, the real speed coincides with the measured speed and follows the reference speed.

Four simulation cases, including all healthy sensors, speed sensor fault, A-phase current sensor fault, and B-phase current sensor fault, were performed to evaluate the performance of the proposed method.

**Figure 7:**
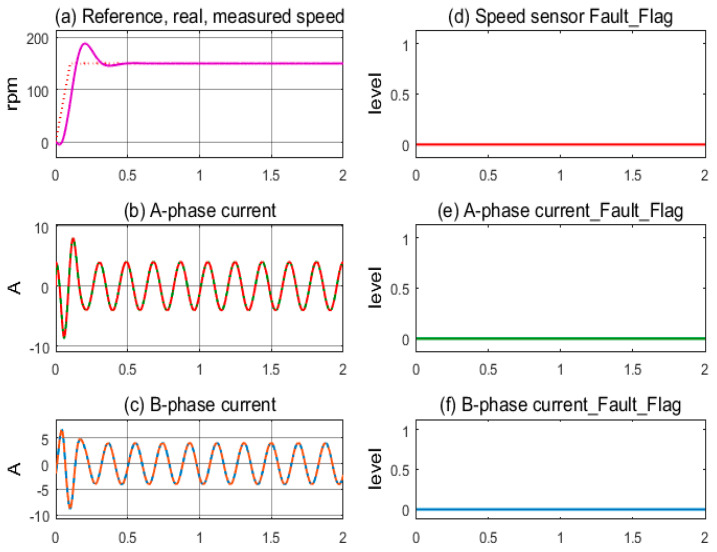
Sensor signals and fault indication flags according to the healthy condition.

**Figure 8:**
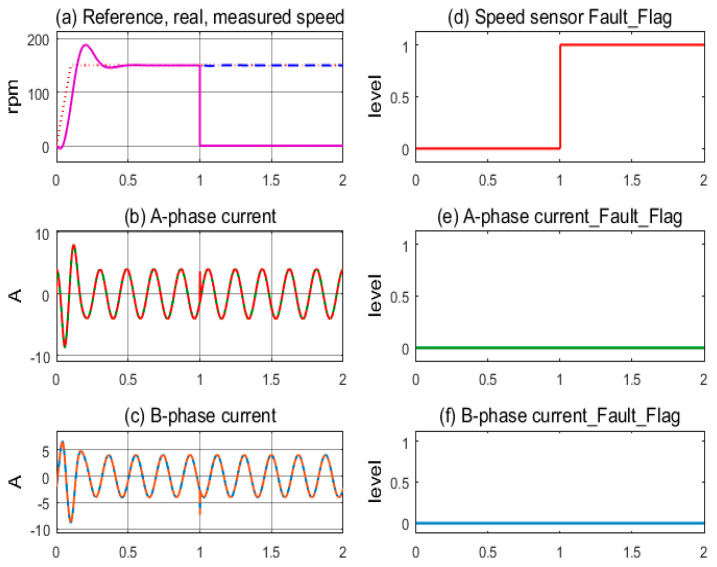
Sensor signals and fault indication flags according to speed sensor fault condition.

The first simulation, in Figure 7, demonstrates the stable operation of the motor drive system corresponding to the healthy sensors mode. The measured speed from the sensor and the motor’s actual speed coincide and follow the reference speed after overcoming the transient period; see Figure 7a. The sinusoidal currents of A-phase and B-phase sensors exhibit stability, as shown in Figure 7b,c. The sensor fault indicating flags are kept low, corresponding to the healthy state, as shown in Figure 7d–f. In Figure 8, the next simulation corresponds to the speed sensor fault occurring at 1.0 s in operation. The measured speed from the sensor and the motor’s actual speed coincide until 1.0 s, when the speed sensor fault occurs and the feedback signal is lost, resulting in the value transfer to the controller being zero, as shown in Figure 8a. The motor current fluctuates for a short time, causing the control command of the FOC controller to be chaotic, as shown in Figure 8b,c. The speed sensor’s fault flag is immediately pushed higher while the other two remain low; see Figure 8d,e. The FTC function is activated when the fault flag is high; the estimated speed immediately replaces the signal from the speed sensor. As a result, the IMD system maintains stable operation under the speed sensorless mode.

**Figure 9:**
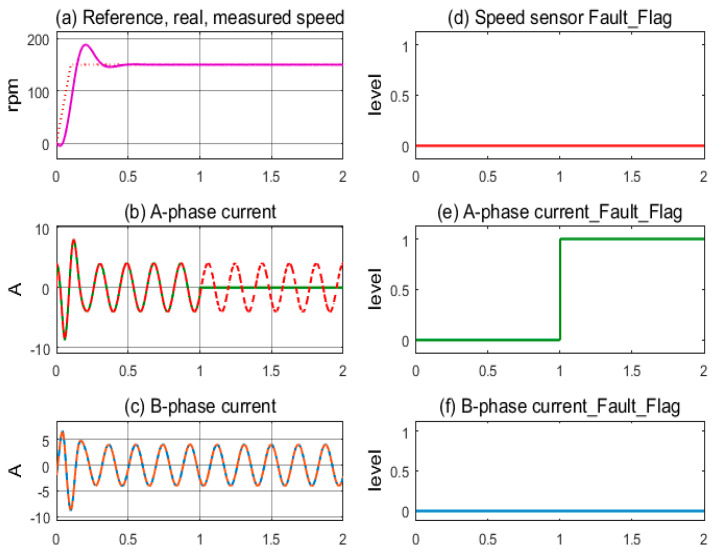
Sensor signals and fault indication flags according to the A-phase current sensor fault condition.

The simulation corresponds to the current sensor fault at the A-phase occurring at 1.0 s in operation. IMD operates stably with full sensor mode until 1.0 s, as shown in Figure 9a. The feedback of the A-phase current signal is lost, and its value to the controller is zero, while the B-phase current is still operating normally; see Figure 9b,c. The A-phase current sensor’s fault flag is immediately pushed higher while the other two remain low; see Figure 9d–f. The FTC function is activated, and the virtual currents immediately replace the current sensor signal. The induction motor still maintains stable operation under the current sensorless mode.

**Figure 10:**
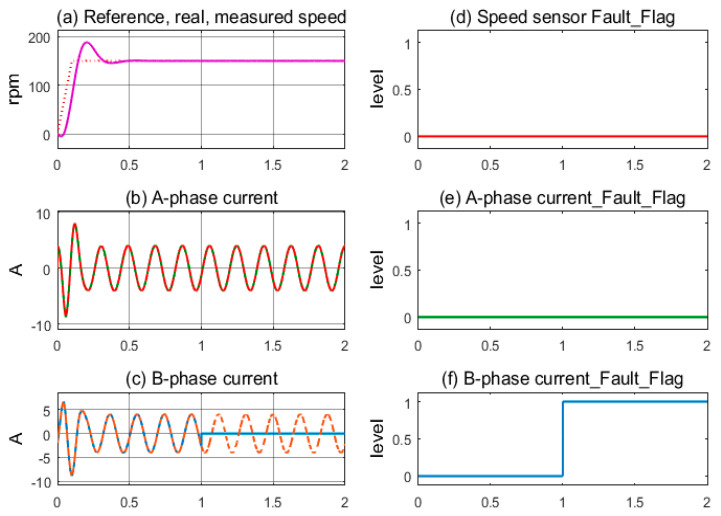
Sensor signals and fault indication flags according to B-phase current sensor fault condition.

The final simulation is carried out according to a current sensor fault in the B-phase, which also occurs at 1.0 s. The operation with full sensor mode is maintained until 1.0 s, as shown in Figure 10a, and then the signal current of the B-phase loses, and the A-phase current still keeps sine form; see Figure 10b,c. The B-phase current sensor’s fault indication flag is immediately pushed higher, while the other two remain low; see Figure 10d–f. Similar to the fault of the A-phase current sensor case, the FTC function is activated, and the current sensors’ incorrect signal is immediately replaced with the virtual currents. Stable operation is maintained under the current sensorless mode. The summary results of the proposed method are presented in Table 2.

In virtual signal generation techniques for the comparison diagnosis method, the estimated current is typically calculated based on the dynamic model of the induction motor with the measured rotor speed as an input, similar to the estimated speed calculated with the input as the measured currents. As a result, methods for diagnosing sensor faults based only on the comparison algorithm between the measured and estimated signals only apply to one current or speed sensor fault type. Therefore, the novelty of the proposed method based on the comparison algorithms between current space vectors is the performance improvement for the comparison diagnosis methods when it can determine the total sensor failures of both the current and the speed sensors. An evaluation between diagnosis methods based on comparative algorithms is presented in Table 3.

The fault diagnosis method correctly detected all three fault cases corresponding to the three sensors in the IMD system; there was absolutely no confusion between the fault types. The proposed method has proven highly reliable in detecting sensor failures through the simulation results.

## 4. Conclusions

A sensor fault diagnosis method based on the machine model is proposed to detect incorrect signals of the sensors in the IMD. The advantage of the proposed method is the improvement of the diagnosis technique based on the comparison algorithm, which has the ability to detect failures of many sensor types. Three current space vectors, measured, and virtual currents are used for fault diagnosis. A sequence is designed to determine the faults according to the rule of checking whether a sensor fault has occurred, determining which sensor type is faulty, and precisely locating the defective sensor. Three sensor failure cases corresponding to signal loss failures were simulated to check the operation of the proposed diagnostic technique. The results were very positive; the FTC function worked quickly and accurately in all three cases, ensuring the continuous operation of the IMD and giving accurate indications to the faulty sensor. The repair and replacement of these faulty sensors will be carried out at the appropriate time in the operating mode. In the future, this method can be integrated with many diagnosis techniques to expand the ability to diagnose more types of signal failure and learn to predict new types of faults in the industrial process.

## Figures and Tables

**Table 1:** Diagnosis function.

Flag Status	Sensor Status	Output
F*_w_* = 0, F*_ia_* = 0, F*_ib_* = 0	Healthy	*ω_m_*, *i_Sα_*, *i_Sβ_*
F*_w_* = 1, F*_ia_* = 0, F*_ib_* = 0	Speed sensor failure	*ω_est_*, *i_Sα_*, *i_Sβ_*
F*_w_* = 0, F*_ia_* = 1, F*_ib_* = 0	A-phase sensor failure	*ω_m_*, *i_Sαest_*, *i_Sβest_*
F*_w_* = 0, F*_ia_* = 0, F*_ib_* = 1	B-phase sensor failure	*ω_m_*, *i_Sαest_*, *i_Sβest_*

**Table 2:** Results summary of the proposed FTC method.

Status of the Sensors			Flag Status			Accurate Signals
Speed Encoder	A-Phase Current	B-Phase Current	Fw	Fia	Fib	*ω_m_*, *ω_est_*, *i_Sα_*, *i_Sβ_*, *i_Sαest_*, *i_Sβest_*
Healthy	Healthy	Healthy	0	0	0	*ω_m_*, *i_Sα_*, *i_Sβ_*
Faulty	Healthy	Healthy	1	0	0	*ω_est_*, *i_Sα_*, *i_Sβ_*
Healthy	Faulty	Healthy	0	1	0	*ω_m_*, *i_Sαest_*, *i_Sβest_*
Healthy	Healthy	Faulty	0	0	1	*ω_m_*, *i_Sαest_*, *i_Sβest_*

**Table 3:** Evaluation of FTC methods based on comparison algorithms.

FTC Method	Applied to Current Sensors	Applied to Speed Sensor	Accurate Faulty Sensor Location	Risk of the Defective Sensor Type Misdiagnosis
LO combining axes transformation [6]	Yes	No	Yes	Yes
Estimated current combining TDO [7]	Yes	No	Yes	Yes
Dual-torque model [9]	Yes	No	Yes	Yes
SMO and SEPLL [13]	Yes	No	Yes	Yes
Current space vector (proposed method)	Yes	Yes	Yes	No

## Data Availability

The data used to support the findings of this study are available from the corresponding author upon request.

## Appendix A

**Table A1:** Mathematical symbols.

Symbols	Names
$\Psi_{S}^{S},\Psi_{R}^{S}$	Stator/rotor flux vector
$i_{S}^{S},i_{R}^{S}$	Stator/rotor current vector
$u_{S}^{S}$	Stator voltage vector
$R_{S},R_{R}$	Stator/rotor resistance
$L_{S},L_{R},L_{m}$	Stator/rotor/magnetizing inductance
$\omega_{r}=p\omega_{m}$	Rotor speed
$\omega_{m}$	Mechanical rotor speed
p	Number of pole pairs
γ	Rotor flux angle

## References

[1] Rahman K., Rahman S., Bhaskar M.S., Iqbal A., Khandakar A., Tariq M., Alamri B. (2022). Field-oriented control of five-phase induction motor fed from space vector modulated matrix converter. IEEE Access.

[2] Mustafa A., Khaled A., Mehrdad E., Aydemir A. (2020). Direct torque control versus indirect field-oriented control of induction motors for electric vehicle applications. Eng. Sci. Technol..

[3] Gouichiche A., Safa A., Chibani A., Tadjine M. (2020). Global fault-tolerant control approach for vector control of an induction motor. Int. Trans. Electr. Energy Syst..

[4] Yu W., Zhao C. (2020). Broad convolutional neural network based industrial process fault diagnosis with incremental learning capability. IEEE Trans. Ind. Electron..

[5] Li W., Feng G., Li Z., Toulabi M.S., Kar N.C. (2021). Extended Kalman filter based inductance estimation for dual three-phase permanent magnet synchronous motors under the single open-phase fault. IEEE Trans. Energy Convers..

[6] Azzoug Y., Pusca R., Sahraoui M., Ammar A., Ameid T., Romary R., Cardoso A.J. (2021). An active fault-tolerant control strategy for current sensors failure for induction motor drives using a single observer for currents estimation and axes transformation. Eur. J. Electr. Eng..

[7] Das S., Manohar M. (2023). A Resilient Current Sensor Fault-Tolerant Strategy for Vector-Controlled Induction Motor Drive. IEEE J. Emerg. Sel. Top. Power Electron..

[8] Ebrahimi S.H., Choux M., Huynh V.K. Diagnosis of Sensor Faults in PMSM and Drive System Based on Structural Analysis. Proceedings of the IEEE International Conference on Mechatronics (ICM).

[9] Li Y., Gong P. (2023). Fault-Tolerant Control of Induction Motor with Current Sensors Based on Dual-Torque Model. Energies.

[10] Zuo Y., Ge X., Chang Y., Chen Y., Xie D., Wang H., Woldegiorgis A.T. (2023). Current Sensor Fault-Tolerant Control for Speed-Sensorless Induction Motor Drives Based on the SEPLL Current Reconstruction Scheme. IEEE Trans. Ind. Appl..

[11] Azzoug Y., Sahraoui M., Pusca R., Ameid T., Romary R., Marques Cardoso A.J. (2021). Current sensors fault detection and tolerant control strategy for three-phase induction motor drives. Electr. Eng..

[12] Liu Y., Stettenbenz M., Bazzi A.M. (2019). Smooth Fault-Tolerant Control of Induction Motor Drives with Sensor Failures. IEEE Trans. Power Electron..

[13] Chen Y., Xie D., Zuo Y., Chang Y., Ge X. Current sensor fault-tolerant control for induction motor with speed-sensorless based on SMO and SEPLL. Proceedings of the 24th International Conference on Electrical Machines and Systems (ICEMS).

[14] Nguyen T.X., Nguyen M.C.H., Tran C.D. (2022). Sensor fault diagnosis technique applied to three-phase induction motor drive. Bull. Electr. Eng. Inform..

[15] Tran C.D., Kuchar M., Sobek M., Sotola V., Dinh B.H. (2022). Sensor Fault Diagnosis Method Based on Rotor Slip Applied to Induction Motor Drive. Sensors.

[16] El Merrassi W., Abounada A., Ramzi M. (2021). Advanced speed sensorless control strategy for induction machine based on neuro-MRAS observer. Mater. Today Proc..

[17] Yan X., Cheng M. (2022). An MRAS Observer-Based Speed Sensorless Control Method for Dual-Cage Rotor Brushless Doubly Fed Induction Generator. IEEE Trans. Power Electron..

[18] Sun X., Zhang Y., Tian X., Cao J., Zhu J. (2022). Speed sensorless control for IPMSMs using a modified MRAS with gray wolf optimization algorithm. IEEE Trans. Transp. Electrif..

[19] Agrawal G., Mohan H., Pathak M. Improved Speed Sensorless Control of Induction Motor Drive Using Artificial Neural Network. Proceedings of the 2nd International Conference on Power Electronics & IoT Applications in Renewable Energy and its Control (PARC).

[20] Zuo Y., Ge X., Zheng Y., Chen Y., Wang H., Woldegiorgis A.T. (2022). An adaptive active disturbance rejection control strategy for speed-sensorless induction motor drives. IEEE Trans. Transp. Electrif..

[21] Tran C.D., Brandstetter P., Nguyen M.C.H., Ho S.D., Pham P.N., Dinh B.H. (2021). An Improved Current-Sensorless Method for Induction Motor Drives Applying Hysteresis Current Controller. Indones. J. Electr. Eng. Inform. (IJEEI).

[22] Tran C.D., Nguyen T.X., Nguyen P.D. (2021). A field-oriented control method using the virtual currents for the induction motor drive. Int. J. Power Electron. Drive Syst. (IJPEDS).

[23] Adamczyk M., Orlowska-Kowalska T. (2019). Virtual Current Sensor in the Fault-Tolerant Field-Oriented Control Structure of an Induction Motor Drive. Sensors.

[24] Azzoug Y., Sahraoui M., Pusca R., Ameid T., Romary R., Marques Cardoso A.J. A Single Observer for Currents Estimation in Sensor’s Fault-Tolerant Control of Induction Motor Drives. Proceedings of the International Conference on Applied Automation and Industrial Diagnostics (ICAAID).

[25] Azzoug Y., Sahraoui M., Pusca R., Ameid T., Romary R., Marques Cardoso A.J. (2020). High-performance vector control without AC phase current sensors for induction motor drives: Simulation and real-time implementation. ISA Trans..

